# Temporal and Spatial Evolution Characteristics of Disturbance Wave in a Hypersonic Boundary Layer due to Single-Frequency Entropy Disturbance

**DOI:** 10.1155/2014/517242

**Published:** 2014-07-06

**Authors:** Zhenqing Wang, Xiaojun Tang, Hongqing Lv, Jianqiang Shi

**Affiliations:** College of Aerospace and Civil Engineering, Harbin Engineering University, Harbin 150001, China

## Abstract

By using a high-order accurate finite difference scheme, direct numerical simulation of hypersonic flow over an 8° half-wedge-angle blunt wedge under freestream single-frequency entropy disturbance is conducted; the generation and the temporal and spatial nonlinear evolution of boundary layer disturbance waves are investigated. Results show that, under the freestream single-frequency entropy disturbance, the entropy state of boundary layer is changed sharply and the disturbance waves within a certain frequency range are induced in the boundary layer. Furthermore, the amplitudes of disturbance waves in the period phase are larger than that in the response phase and ablation phase and the frequency range in the boundary layer in the period phase is narrower than that in these two phases. In addition, the mode competition, dominant mode transformation, and disturbance energy transfer exist among different modes both in temporal and in spatial evolution. The mode competition changes the characteristics of nonlinear evolution of the unstable waves in the boundary layer. The development of the most unstable mode along streamwise relies more on the motivation of disturbance waves in the upstream than that of other modes on this motivation.

## 1. Introduction

Because hypersonic flow is far more complex than low speed flow (low Mach numbers flow and incompressible flow), the prediction of hypersonic boundary layer transition has become more difficult, which makes the transition mechanism of hypersonic boundary layer unclear. There is a lack of mature model for making accurate predictions of hypersonic boundary layer transition. The prediction of transition is complex due to the fact that the shear flow is very difficult to determine. The difficulty of transition prediction also is caused by the flow accompanied by various initial conditions and various freestream conditions [1]. The drag and heat transfer of aircraft in the laminar state are significantly different from those in the turbulent state. The flow separation, drag, and thermal load are directly related to the flow state of boundary layer. Therefore, the investigations on the mechanism transition of hypersonic compressible flows have important theoretical and engineering significance for hypersonic vehicles design. The mechanisms about how the initial disturbance waves are induced in the boundary layer and how the unstable disturbance waves in the boundary layer are motivated are not fully understood.

In order to understand the stability characteristic of hypersonic boundary layer, scholars have done much on this problem by experiments. Potter [2] experimentally investigated the transition of flat-plate boundary layer and the stability of hypersonic tunnel flow. It is found that the disturbance amplitude obtained by experiments agrees well with the linear stability theory (LST) result when the freestream Mach number is less than 2.2. There are similar qualitative results between experiment and LST when freestream Mach number is larger than 8.5. A series of experimental studies focusing on low Reynolds number tunnel are performed by Dietz [3] for investigating boundary layer receptivity with specific disturbances. Stetson and Kimmel [4–6] conducted a series of experiments on the hypersonic blunt cone boundary layer, including the effects of wall temperature, angle of attack, Reynolds number, and freestream velocity on the stability of hypersonic boundary layer. In recent years, with the development of computational fluid dynamics (CFD) and computer performance, a series of numerical studies are performed to understand how the freestream disturbance wave interacts with shock wave and boundary layer, how the unstable waves are induced, how the induced disturbance waves interact with shock wave and boundary layer, and how they evolve [7–11]. Johnson [7] studied the receptivity of a zero pressure gradient boundary layer under different freestream disturbances with simple waveform and found only a minority of freestream disturbance waves play a significant effect on the receptivity of zero pressure gradient boundary layer. Ricco and Wu [8] investigated the response of boundary layer to freestream vertical disturbance and the sensitivity of boundary layer to vortical disturbances. Ma and Zhong [9, 10] investigated the supersonic and hypersonic boundary receptivity to freestream disturbances. The effects of bluntness on the stability characteristics of hypersonic boundary layers have been studied numerically by Zhong and Ma [11, 12]. Among these investigations, most are based on the continuous small disturbance, such as freestream small disturbance and wall surface disturbance. The receptivity of hypersonic boundary layer to various disturbances and the effects of disturbance wave on laminar-turbulent transition are researched to gain the influence mechanism of different disturbance waves to the stability of hypersonic boundary layer in these investigations. These investigations indicate that the type of disturbance wave, the flow conditions, and so forth have a major impact on the interaction between disturbance wave and hypersonic flowfield, the evolution of disturbance waves in the hypersonic boundary layer, and the stability characteristics of boundary layer. The stability of transient boundary layer has been systematically analyzed in many researches.

Among these investigations, only the period phase of flow under disturbance has been investigated in most of these researches [13]. The disturbance waves' evolution along the streamwise (spatial evolution) in the hypersonic boundary layer has been studied in detail. In fact, there are three phases in the process of the interaction between freestream disturbance waves and flowfield, namely, the response phase, the period phase and the ablation phase. It should be mentioned that the period phase is the one in which the flowfield reaches time-period state [13, 14] under the interaction between freestream disturbance and flowfield. The response phase is the initial phase after the flowfield is subject to freestream disturbance. That is to say, it refers to the phase from the state in which the flowfield is subject to freestream disturbance to its time-period state. The ablation phase is the decaying process of disturbance wave in the boundary layer after the freestream disturbance being terminated. However, few researches focused on the three phases of boundary layer disturbance waves' evolution, namely, from response phase to period phase and finally to ablation phase, when the hypersonic flowfield is subject to freestream disturbance wave. That is, the temporal evolution of disturbance wave modes in the boundary layer is seldom conducted. Actually, the generation and evolution in the period phase coincided with the response of boundary layer to disturbance. Meanwhile, when the introduction of initial disturbance is terminated, the attenuation characteristics of disturbance wave in the boundary layer are beneficial to understand the stability mechanism of boundary layer. Therefore, to understand the generation and the evolution mechanism of disturbance wave in the boundary layer, it is necessary to investigate which disturbance wave is induced, how the boundary layer responds to initial disturbance, and how disturbance wave will evolve in the boundary layer after the introduction of initial disturbance is terminated. To put it simply, both the temporal evolution and spatial evolution of disturbance wave modes in the boundary layer should be investigated.

In the present paper, based on hypersonic unstable flow over a blunt wedge under freestream entropy disturbance, the response of hypersonic boundary layer to freestream entropy disturbance and the interaction between freestream disturbance and hypersonic flowfield are investigated. Both the temporal evolution and spatial evolution of disturbance wave modes in the boundary layer are discussed through Fourier frequency spectral analysis.

## 2. Solution Algorithm

For direct numerical simulation, the governing equations are the two-dimensional Navier-Stokes (N-S) equations. The governing equations can be written in the following form in the Cartesian coordinates:

(1) $$\begin{array}{c} \frac{\partial U}{\partial t}+\frac{\partial F_{i}}{\partial x_{i}}+\frac{\partial F_{vi}}{\partial x_{i}}=0,\;\left(i=1,2\right), \end{array}$$

where the variables **U**, **F**_*i*_, and **F**_*vi*_ in the N-S equations are the vectors terms, convective terms, and viscous flux terms, respectively. While the variables can be expressed as follows:

(2) $$\begin{array}{c} U=\left[\rho ,\rho u_{1},\rho u_{2},\rho e\right],\\ F_{i}=\left[\begin{array}{c} \rho u_{i}\\ \rho u_{1}u_{i}+P\delta_{1i}\\ \rho u_{2}u_{i}+P\delta_{2i}\\ \left(\rho e+P\right)u_{i} \end{array}\right],\\ F_{vi}=\left[\begin{array}{c} 0\\ \tau_{1i}\\ \tau_{2i}\\ \tau_{ij}u_{j}-q_{i} \end{array}\right],\\ P=\rho RT, \end{array}$$

where the variables  *ρ*, *u*_*i*_, *e*, *δ*_*ij*_, *τ*_*ij*_, *P*, *T*, and *k* are density, velocity, total energy, Kronecker symbol, shear stress, pressure, temperature, and heat conductivity coefficients, respectively. The viscosity coefficients are calculated according to the Sutherland law with the assumption of zero bulk viscosity. The total energy *e* is gained by

(3) $$\begin{array}{c} e=c_{v}T+\frac{1}{2}u_{k}^{2}. \end{array}$$

The heat conductivity coefficients *k* are determined by assuming a constant Prandtl number Pr; it can be expressed as

(4) $$\begin{array}{c} k=\frac{\mu c_{p}}{\text{Pr}}, \end{array}$$

where *μ*, *c*_*v*_, and *c*_*p*_ are the viscosity, the specific heat at constant volume, and the specific heat at constant pressure, respectively.

**Figure 1:**
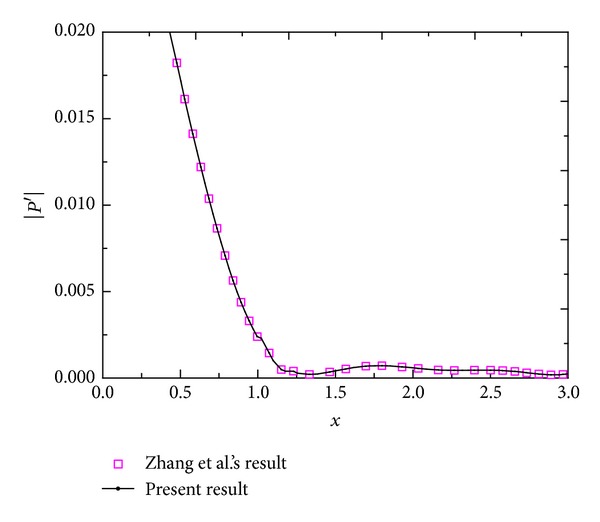
Amplitudes of the second harmonic mode of pressure disturbance in the boundary layer are compared with Zhang et al.'s result [21, 22].

Since finite difference method can be easily applied to the simulations of flowfiled existing complex geometries, it has been widely employed in the DNS of unsteady flows, especially compressible unsteady flows [15–18]. Because central difference schemes only introduce phase errors but no dissipative errors in numerical solutions, the schemes have been widely implemented in direct numerical simulation [15]. However, they are not robust enough in the simulations of convection dominated flow [18]. To provide adequate accuracy level for DNS, high-order schemes are required. However, the higher-order numerical scheme generally introduces the numerical oscillatory behavior near the discontinuity [19], while weighted essentially nonoscillatory (WENO) scheme [20] can be used to suppress the oscillatory behavior near the discontinuities or high gradient regions. In addition, upwind schemes show strong robustness in hypersonic flow simulation even when they are made high-order accurate [18]. Therefore, the governing equations are solved by using the 6th order center difference scheme and 5th order upwind WENO scheme for viscous flux terms and convection terms, respectively. Meanwhile, to maintain adequate time accuracy, a third-order, total variation diminishing Runge-Kutta scheme [19] is used for time integration. To validate the numerical program employed in this paper, a hypersonic unsteady flow over a blunt wedge with 5° half-wedge-angle (Zhang et al's numerical model [21]) under the action of freestream disturbance wave is solved in our previous investigation [22]. The amplitudes of the second harmonic mode of pressure disturbance in the boundary layer are compared with Zhang et al.'s result [21], as shown in Figure 1. Figure 1 shows that the numerical program is reliable.

## 3. Computational Conditions

Computational model is a hypersonic flow over a wedge with blunt noses. Freestream conditions and extrapolation are employed at the upstream boundary and the outflow, respectively. Symmetry conditions are used at *y* = 0. No-slip and constant temperature conditions are imposed on the wall. The variables *ρ*_*∞*_, *P*_*∞*_, *r*/*u*_*∞*_, *R*, *u*_*∞*_/*r*, and *u*_*∞*_ are used for nondimensionalizing density *ρ*, pressure *P*, time *t*, length, frequency *f*, and velocity, respectively. Subscripts “*∞*” denote freestream condition.

After the steady flowfield without disturbance is computed, the entropy disturbance with single mode is introduced to the upstream boundary from *t* = 0 to *t* = 48. The entropy disturbances [21, 23] that impinge on the upstream boundary are taken to

**Figure 2:**
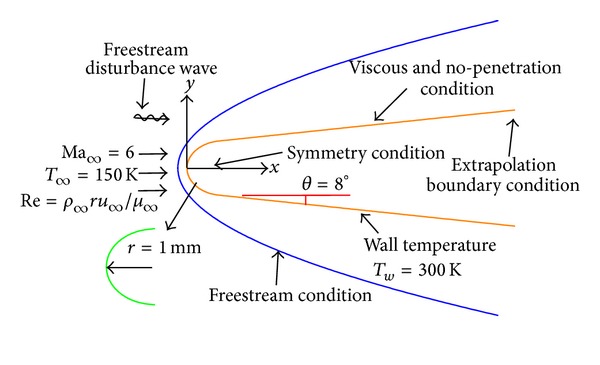
Computational model.

**Figure 3:**
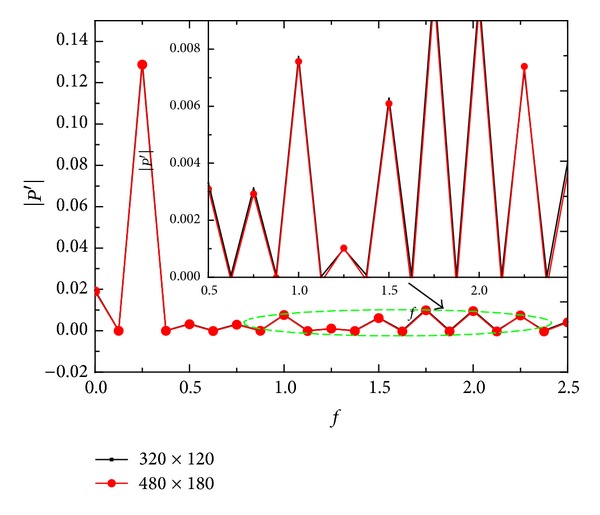
The pressure disturbance mode obtained under two grid conditions.

(5) [u′v′p′ρ′]T  =[000AMa]Tei(βx−(γ·Re/106)t+(π/2)),

where the variables *u*′, *v*′, *P*′, and *ρ*′ are the disturbance values of the velocity along axis *x*, and velocity along axis *y*, pressure and density, respectively; *A*, *β*, *γ*, Ma, and *Re* are amplitude, wave number, generalized frequency freestream Mach number, and Reynolds number, respectively. The superscripts “′” that used in the follow denote disturbance values which are obtained by the variables value of instantaneous flow minus the variables value of the local steady base flow. The computational conditions and flow parameters for the model are shown in Figure 2 and Table 1. The variable *η* is the angle of attack. A 300 × 120 grid is used for the steady and unsteady calculations. The grid lines stretching method is used in wall-normal directions to cluster more points inside the boundary layer where strong shear flow exists. The grid lines are also stretched in the streamwise for maintaining a good resolution in strong shock wave regions. The mesh grid density which introduces the present computations matches that in the investigations with similar computational model conducted by Duan et al. [18], Zhang et al. [21], and Prakash et al. [24]. It should be pointed out that the stretching method is employed in the numerical calculation of Figure 1. Since the amplitudes of disturbance in the boundary layer obtained by the stretching method agree well with Zhang et al's result [21], it can be believed that the stretching method is available. Meanwhile, to evaluate the reliability of the grid density, the simulations with two kinds of grid (300 × 120 and 450 × 180) are conducted under the condition employed in this paper. The pressure disturbance mode in boundary layer obtained under the two conditions is shown in Figure 3. From Figure 3, it can be seen the difference of the pressure disturbance mode in boundary layer under the two conditions is tiny, which indicates the grid density in this paper, especially the one in the vicinity of the boundary layer (near the wall) is reliable.

## 4. Results and Discussions

### 4.1. Interaction between Freestream Entropy Disturbance and Hypersonic Flow

**Figure 4:**
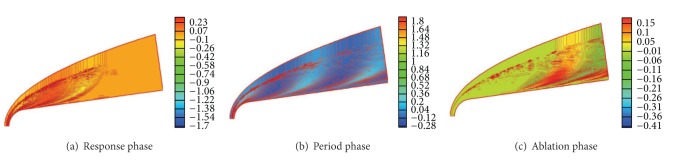
Contours of entropy disturbance in different phases.

Figure 4 shows the contours of entropy disturbance En′(*x*, *y*, *t*) in different phases (response phase, period phase, and ablation phase) under freestream entropy wave with the amplitude *A* = 8 × 10^−3^. The contours in the response phase, period phase, and ablation phase are shown in Figures 4(a), 4(b), and 4(c), respectively. The time for the Figures 4(a), 4(b), and 4(c) is *t* = 6.0, *t* = 48, and *t* = 54, respectively. Figure 4 shows the strong interaction between the freestream disturbance wave and the bow shock wave and the difference between the inside boundary and the outside boundary layer. The simulation result shows that, under the action of shock wave, the disturbance amplitude is amplified obviously considering the freestream wave amplitude *A* = 8 × 10^−3^. This means that the strong disturbance waves are generated owing to the interaction between freestream entropy disturbance and shock wave. From Figure 4(c), it can be seen that the residual disturbance exists near shock wave in the upstream in the ablation phase when freestream disturbance is terminated. It is attributed to the fact that there still remain reflected waves between shock wave and wall surface after halting disturbance in ftreestream, and the reflected wave is enlarged by shock wave in each reflection [25]. The enlarged wave will be significantly dissipated when leaving shock wave, which makes the residual disturbance exists only near shock wave in upstream [22].

**Figure 5:**
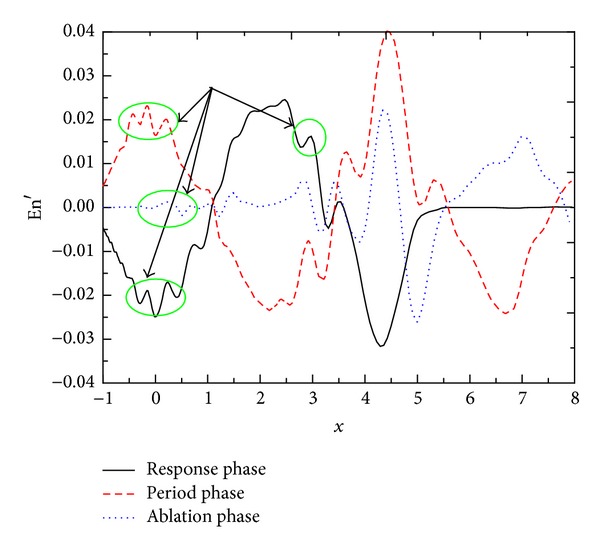
Distribution of entropy disturbance En′(*x*, *y*, *t*) on wall.

Figure 5 shows the distribution of entropy disturbance En′(*x*, *y*, *t*) on wall surface in the response phase, period phase, and ablation phase under freestream entropy disturbance with the amplitude *A* = 8 × 10^−3^. After the action of shock wave, the disturbance waves enter boundary layer and change the state entropy of boundary layer. It can be seen from Figure 5 that the distribution of entropy disturbance on wall surface is changed sharply under freestream disturbance wave. It clearly indicates that the disturbance wave in the boundary layer has not propagated to downstream and there is no entropy disturbance on wall. The distribution of disturbance on wall in the response phase is rather different from that in the period phase. In the ablation phase, the amplitude of entropy disturbance on wall decays rapidly in the upstream due to the loss of disturbance excitation in freestream. Before the following discussion, it should be mentioned that the disturbance waves are induced after the interaction between freestream disturbance and shock wave; most of the induced waves propagate from upstream to downstream, which is called the mainstream disturbance in this paper. However, as discussed earlier, a part of the induced waves will move back and forth between shock wave and nose [25], which is the reflected wave. From Figure 5, in the response phase, period phase and ablation phase, due to the action of reflected wave, there are fluctuations at the distribution curve of entropy disturbance on wall, as the circular mark shown in Figure 5. It also can be seen that the mainstream disturbance wave affects more entropy disturbance than reflected wave. Owing to that (1) it is believed that only a small part of the induced waves reflects between the shock wave and nose; (2) the viscidity dissipation for reflected wave is larger than that for mainstream wave due to the fact that the propagation path of the former is larger than the latter. Figure 5 shows the amplitude of entropy disturbance on nose wall in the ablation phase tends to be zero, which is significantly smaller than in both the response phase and period phase. This is the result of the loss of freestream disturbance; only the weak reflected waves exist in the nose boundary layer, which have been severely dissipated. Their influence on the entropy on wall is rather limited.

### 4.2. Analysis of Nonlinear Evolution of Disturbance Wave in the Boundary Layers

**Figure 6:**
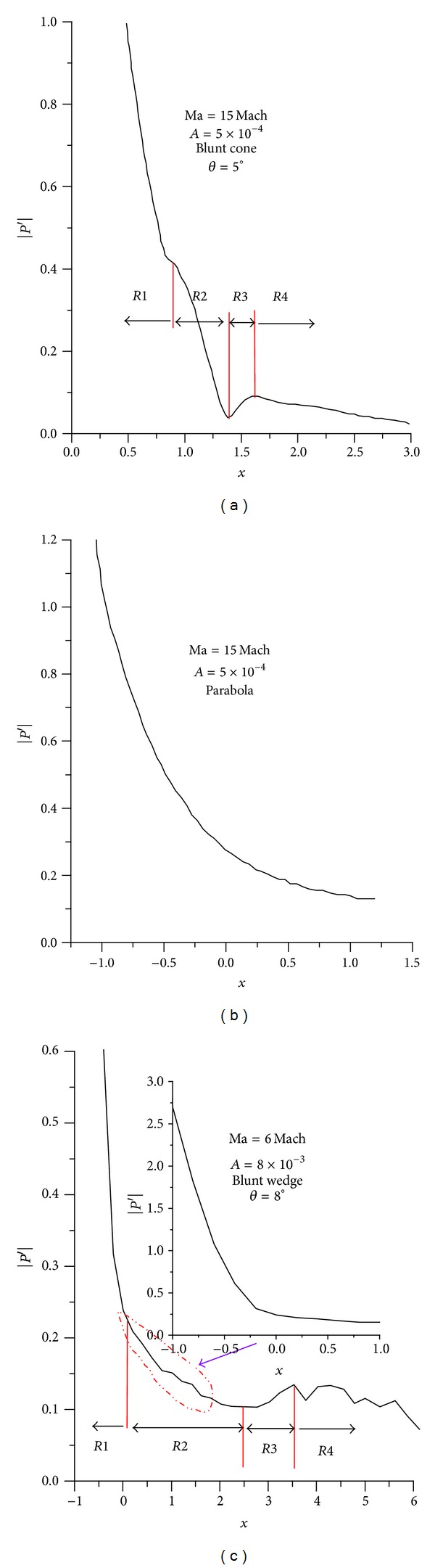
Fourier amplitudes of pressure disturbance in boundary layer are compared with literatures [21, 23].

Since the evolution of disturbance mode in boundary layer has a significant effect on boundary layer stability [1, 22, 26], the nonlinear evolution of disturbance wave in the boundary layers is investigated by analyzing the pressure disturbance on wall. Different locations on the wall surface are selected to record the disturbance value of aerothermodynamics parameter in the boundary layer and to explore the temporal and spatial evolution (streamwise evolution) of disturbance waves in the boundary layer. A fitted coordinate *s* corresponding to *x* is employed for representing the curve length from wall location to the stagnation point and the fitted coordinate is the same as that in [25]. The time domain signals of pressure disturbance at different wall locations in both the response phase, period phase, and ablation phase are transformed by Fourier series. The temporal signals of pressure disturbance are decomposed into frequency signals using Fourier transform, which makes the time domain signals into the frequency domain signals.  Figure 6 shows that the distribution of the Fourier amplitudes of pressure disturbance along streamwise in the hypersonic blunt body boundary layer under freestream entropy wave in the period phase is compared with literatures [21, 23]. The computational conditions are included in the figure. Figures 6(a), 6(b), and 6(c) are Zhang et al.'s result [21], Zhong's result, and present result, respectively. As shown in the figure, the Fourier amplitudes of pressure disturbance in the three kinds of the hypersonic blunt body boundary layer under freestream entropy wave have similar change tendency along streamwise. That is to say, it sharply decreases along streamwise in the nose boundary layer and the decreasing rate becomes small in the no-nose boundary layer. However, it can be seen that there is an obvious difference among the three kinds of Fourier amplitude. By comparing Figures 6(a) and 6(c), it can be seen that there are four similar areas, as the marks* R*1,* R*2,* R*3, and* R*4 shown in the figure. In the nose boundary layer (in Figure 6(a),  *x* < 1; in Figure 6(c),  *x* < 0), namely,* R*1 area, the amplitude sharply decreases, the amplitude in* R*2 area decreases slowly and in* R*3 area, it increases obviously and it generally decreases in* R*4 area. The reason why the amplitude remains a decreasing tendency in* R*2 area is that there is expansion wave in the junction region (in Figure 6(a),  *x* = 1; in Figure 6(c),  *x* = 0) between the spherical nose and the straight cone which is caused by the surface curve discontinuity [25]. However, in the no-nose area, the flow will be recompressed [25]. Thus, the decreasing tendency slows. In the* R*3 area, the effect of the expansion waves on the pressure disturbance in the boundary layer decreases with the distance departing from the junction region. The flow recompression obviously increases the amplitude of pressure disturbance. What is worth mentioning is that there is small fluctuation of the amplitude of pressure disturbance in Figure 6(c)'s* R*4 area. It can be believed that the phenomenon is due to the nonlinearity of disturbance evolution caused by the fact that the amplitude of freestream disturbance in this paper is significantly larger than that in literature [21].

**Figure 7:**
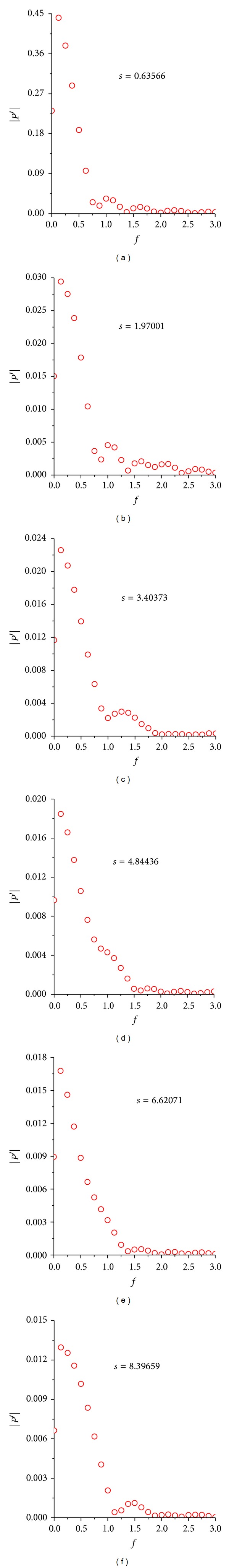
Frequency spectrum analysis of pressure disturbance at different locations in the response phase.

Figure 7 shows the Fourier frequency spectrum analysis of pressure disturbance at different surface locations in the response phase. It is obtained that (1) the Fourier amplitude of pressure disturbance in the nose boundary layer (*s* = 0.63566) is considerably larger than that in the no-nose boundary layer. The Fourier amplitude decreases sharply along streamwise in the nose boundary layer; however the attenuation becomes sharply in the no-nose boundary layer. As discussed in Figure 4, freestream disturbance wave will be enlarged under the action of shock wave; From Figure 7, it is obtained that the effects of normal shock wave are larger than that of oblique shock wave on freestream disturbance. (2) In the response phase, when *s* = 0.63566 (in the boundary layer), the main disturbance modes in the boundary layer are falling in *f* < *f*_3_, which is distributed mainly near fundamental mode (*f*_1_), and the Fourier amplitudes of the modes with frequency larger than *f*_3_  (*f* > *f*_3_) are tiny. It should be mentioned that *f*_*n*_ denotes the *n*th harmonic mode in this paper, namely, *f*_1_ = 0.25, *f*_3_ = 0.75, and *f*_*n*_ = 0.25*n* and the following is the same. As the disturbance wave propagates from upstream to downstream, the percentage of high frequency disturbance wave increases; the frequency range of the main disturbance modes in the boundary layer is expanded to 0 < *f* < *f*_5_, which is distributed mainly near the second harmonic mode (*f*_2_). These indicate that the harmonic modes even high frequency disturbance waves are induced under the interaction between freestream disturbance and shock wave as well as hypersonic boundary layer. In the response phase, the blunt wedge boundary layer is dominated by the modes near fundamental mode and second harmonic mode.

**Figure 8:**
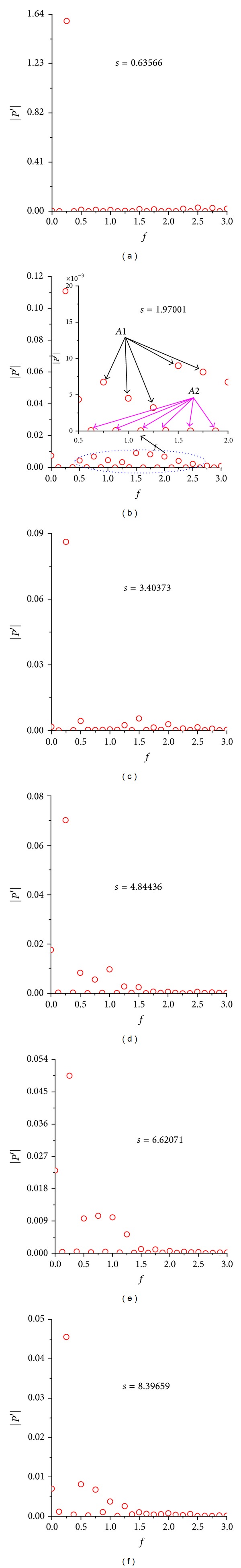
Frequency spectrum analysis of pressure disturbance at different locations in the period phase.

Figure 8 shows the Fourier frequency spectrum analysis of pressure disturbance at different surface locations in the period phase. It can be found that, both in the nose boundary and in the no-nose boundary layer, the Fourier amplitude of the fundamental mode is significantly larger than that of the other modes in the period phase; the fundamental mode is the dominant mode in the boundary layer. Similar to the case in the response phase, the Fourier amplitude of pressure disturbance in the nose boundary layer is considerably larger than that in the no-nose boundary layer in period phase. In the nose boundary layer, the fundamental mode is the only main disturbance mode since the Fourier amplitude of the other modes is very tiny and the effect of the other modes can be neglected. With the disturbance evolving from the upstream to the downstream, the high frequency components increase quickly and the amplitude of the fundamental mode is restrained to grow. It should be mentioned that the high frequency components are mainly distributed near harmonic frequency modes (*f*_*n*_, *n* is integer), as the mark* A*1 shown in Figure 8, and the Fourier amplitudes of the other high frequency modes are still tiny, as the mark* A*2 shown in Figure 8. It can be clearly observed that the proportion of the modes in the range from *f*_2_ to *f*_10_ appear different levels of increases when *s* from 0.63566 to 1.97001. When *s* = 4.84436, the proportion of the modes of *f*2–10 declines along streamwise rapidly, and the frequency range narrows. When *s* = 8.39659, the main disturbance modes in the boundary layer are mainly distributed near the fundamental mode and the modes in the range from *f*_2_ to *f*_5_ and the other modes are basically filtered out. This implies that (1) the disturbance waves within a certain frequency range will be generated in the transition region between the nose and no-nose boundary layer under the interaction between freestream disturbance and shock wave as well as boundary layer. (2) With the disturbance evolving from the upstream to the downstream, most of disturbance waves in the boundary layer decrease, and only special frequency ranges (*f*_1_–*f*_5_) of unstable wave exist in the downstream boundary layer, indicating mode competition exists during the disturbance wave evolution along streamwise in the boundary layer. By comparing Figure 7 with Figure 8, it can be seen that the frequency spectrum of pressure disturbance in the period phase is remarkably different from that in the response phase. The frequency range of main disturbance modes in the boundary layer in the response phase ranges from 0 to *f*_4_; while the main disturbance modes in the boundary layer are fundamental modes and the amplitudes of harmonic frequency are small in the period phase. This indicates that mode competition exists during the process of changing from response phase into period phase. In the process, the mode competition makes the fundamental mode sharply increases and the other modes slowly increase or are suppressed.

**Figure 9:**
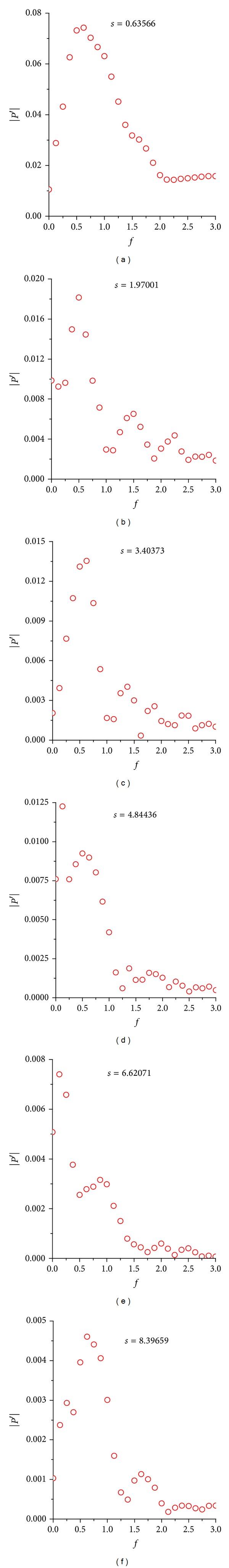
Frequency spectrum analysis of pressure disturbance at different locations in the ablation phase.

Figure 9 shows the Fourier frequency spectrum analysis of pressure disturbance at different surface locations in the ablation phase. As discussed in Figure 8, we know the main disturbance modes in the boundary layer is fundamental mode *f*_1_ and the amplitudes of harmonic frequency (*f*_*n*_, *n* ≥ 2) are small in the period phase. However, it can be clearly observed from Figure 9 that the disturbance mode in the boundary layer is widely distributed in the range from 0 to *f*_12_ in the ablation phase. The proportion of the modes with frequency larger than 0.25 (*f* > 0.25) in the boundary layer will grow by a significant amount during the process of changing from the period phase into the ablation phase although all modes will finally disappear due to the loss of disturbance excitation in freestream. It indicates that, when the freestream disturbance is terminated, the mode with the amplitude of fundamental mode decreases sharply and firstly with the temporal evolution of disturbance wave in the boundary layer; the disturbance energy of fundamental mode is transferred to the modes with frequency larger than the second harmonic frequency, especially the modes in the range from *f*_2_ to *f*_8_ (*f* = 2.0). Meanwhile, from Figure 8, we know that, in the period phase, the disturbance waves are mainly distributed near fundamental mode and harmonic frequency modes (*f*_*n*_, *n* is integer) and the Fourier amplitudes of the other high frequency modes are still tiny. As seen in Figure 9, in the ablation phase, the disturbance waves are widely distributed in the range from *f* = 0 to *f* = 3.0. Namely, the frequency range in the boundary layer in the period phase is narrower than that in the ablation phase, which indicates that a part of the disturbance energy of *f*_*n*_ (*n* is integer) is transferred to other modes. It also can be seen that, due to the transformation of disturbance energy in the boundary layer, when *s* < 4.84436, the dominant mode in the boundary layer is transferred from the fundamental mode to the second harmonic mode during the process of changing from period phase into ablation phase; when *s* > 4.84436, the dominant mode in the boundary layer is transferred from the fundamental mode to near the third harmonic mode during the process of changing from period phase into ablation phase. That is, there are the transformations of the dominant mode in both temporal and spatial evolution of disturbance wave modes in the boundary layer.

**Figure 10:**
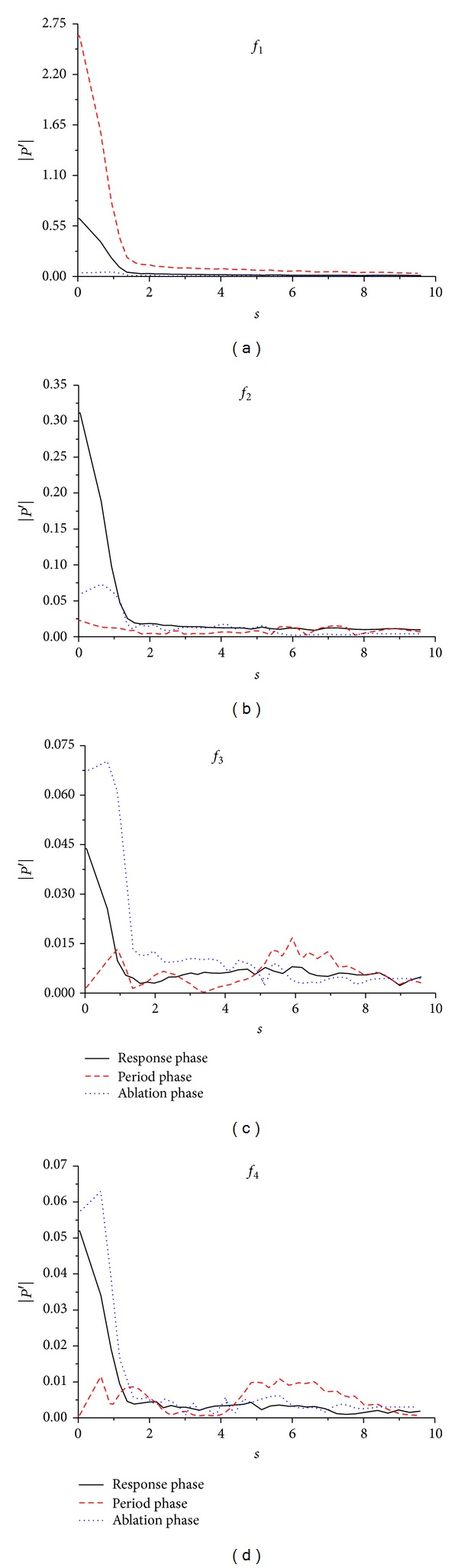
Comparison of pressure disturbances amplitudes in the boundary layer in 3 phases.

Figure 10 shows the comparison of the Fourier amplitudes of different disturbance modes *f*_*n*_ in the boundary layer in 3 phases. It can be seen that the Fourier amplitude of *f*_1_–*f*_4_ in the boundary layer changes along streamwise during the process of flowfield state from response phase to period phase and finally to ablation phase. (1) For fundamental mode, the Fourier amplitude of the fundamental mode in the period phase is larger than that in the response and ablation phase in both the nose boundary layer and the no-nose boundary layer. (2) For the second harmonic mode, when *s* < *π*/2, the Fourier amplitude of the second harmonic mode in the response phase is larger than that in the period and ablation phases. (3) In the nose boundary layer, the Fourier amplitude of the third harmonic mode and the forth harmonic mode in the ablation phase is larger than that in the other two phases. It should be pointed that, when *s* > 5, the Fourier amplitude of the third harmonic mode and the fourth harmonic mode in the period phase is larger than that in the response and ablation phase. (4) As seen in Figure 10, the third and fourth harmonic modes are induced in the nose boundary layer in the response phase; their amplitudes decrease with the flowfield state from the response phase to the period phase, whereas their amplitudes increase with the flowfield state from the period phase to the ablation phase. Namely, in the ablation phase, before the third and fourth harmonic modes decrease with the temporal evolution of disturbance wave in the boundary layer, they increase firstly. Therefore, it can be obtained from Figure 10 that there are mode competitions between different modes in the temporal evolution of disturbance wave.

**Figure 11:**
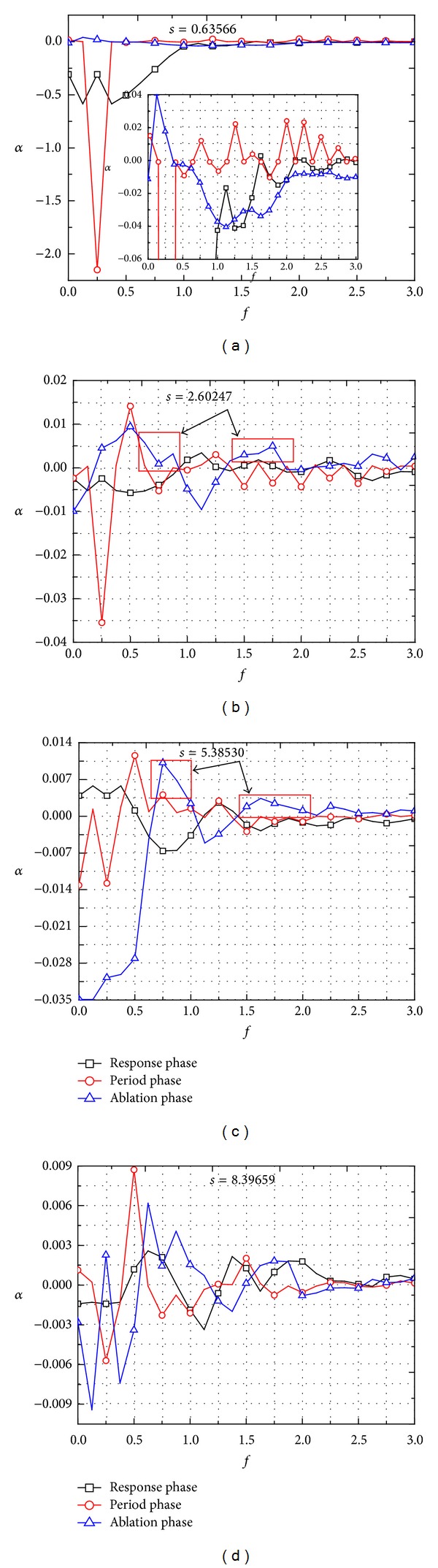
Growth of different frequency disturbances in the boundary layer along streamwise in 3 phases.

Figure 11 shows the growth rate of different frequency disturbances in the boundary layer along streamwise in the three phases, namely, response phase, period phase, and ablation phase, respectively. It can be seen that (1) the main disturbance modes in the nose boundary layer (*s* = 0.63566) decay along streamwise in all the three phases. It is believed that because the bow shock wave changed from the normal shock in nose region to the oblique shock in no-nose region, the former is stronger than the latter. It implies that the disturbance evolution in the nose boundary layer is significantly affected by the shock wave. However, it can be found that the growth of many disturbance modes along streamwise in the no-nose boundary layer is larger than 0. Namely, these modes in the no-nose boundary layer have an increasing trend in the disturbance's evolution along streamwise, indicating that the bow shock no longer plays a leading role in the evolution of the disturbance. (2) When *s* = 2.60247, the modes with the frequency less than 1.0 decrease along streamwise and some modes within the range of *f* ≥ 1.0 increase slowly in the response phase; all the modes except the second harmonic mode and the fifth harmonic mode decrease or basically remain stable along streamwise in the period phase; the fundamental mode, the second harmonic mode, sixth harmonic mode, and the seventh harmonic mode increase along streamwise and the other modes decrease or basically remain stable along streamwise in the ablation phase. This means different phases have different unstable modes in the boundary layer. Namely, the unstable mode in the boundary layer changes with the temporal evolution of disturbance wave in the boundary layer. (3) The growth rate of some modes in the range of frequency *f* > 0.5 in the ablation phase is larger than that in the period and response phase, as the rectangular mark shown in Figure 11. For instance, the growth rate of the modes near *f* = 0.75 and *f* = 1.75 at *s* = 5.38530 in the ablation phase is larger than that in the other two phase. This also means that some modes in the boundary layer are suppressed during response and period phase; mode competition between these modes and the main disturbance modes exists in the temporal evolution of boundary layer disturbance wave, which can change the characteristics of nonlinear evolution of the unstable waves in the boundary layer. (4) In the period phase, in both the nose boundary and the no-nose boundary, the attenuation rates of fundamental mode are significantly higher than that of other modes; in no-nose boundary, the second harmonic mode becomes the mode with the highest growth rate, which is the most unstable mode. It is also seen that, in the downstream boundary layer (*s* = 5.38530, 8.39659), the attenuation rates of the most unstable mode are significantly higher than that of other modes when the boundary layer state changes from period phase to ablation phase. This shows that the development of the most unstable mode along streamwise relies more on the motivation of disturbance waves in the upstream than that of other modes.

## 5. Conclusions

The temporal and spatial nonlinear evolution characteristics of disturbance wave in the hypersonic boundary layer over a blunt wedge due to single-frequency entropy disturbance are proposed in this paper, and some conclusions are drawn.

1. Under the action of freestream entropy wave, the entropy state of boundary layer is changed sharply and the effect of the mainstream disturbance wave on the entropy state in the boundary layer is larger than that of the reflected wave. Under freestream single-frequency entropy wave, the disturbance waves within a certain frequency range are induced in the hypersonic boundary layer in the three phases. The bow shock plays a leading role in the disturbance wave evolution along streamwise in the nose boundary layer, whereas the leading role disappears in the no-nose boundary layer.
2. The disturbance frequency spectrum in the period phase is remarkably different from that in the response and ablation phase. In the period phase, the majority of the disturbance modes in the boundary layer are distributed near fundamental mode and harmonic modes; while in the response and ablation phase, the disturbance modes in the boundary layer are widely distributed in different modes. The frequency range in the boundary layer in the period phase is narrower than that in the response phase and ablation phase. However, the amplitude of boundary layer disturbance waves in the period phase is significantly larger than that in these two phases.
3. The mode competition, dominant mode transformation, and disturbance energy transfer exist among different modes both in the temporal evolution and in the spatial evolution of boundary layer disturbance waves. Mode competition narrows the frequency range of unstable waves in the boundary layer, which changes the characteristics of nonlinear evolution of the unstable waves. The development of the most unstable mode along streamwise relies more on the motivation of disturbance waves in the upstream than that of other modes on this motivation.

## Figures and Tables

**Table 1:** Computational conditions.

*η* (°)	Re	*A *	*β*	*γ*
0	6000	8 × 10^−3^	3.1446 × 10^−4^	50*π*
